# Algorithm-guided personalized pulsed field ablation for persistent atrial fibrillation: a prospective multicentre study with matched external comparison

**DOI:** 10.1093/europace/euag225

**Published:** 2026-08-28

**Authors:** Frédéric Anselme, Decebal Gabriel Lațcu, Théo Méral, Arnaud Savoure, Serge Boveda, Pascal Defaye, Sandrine Venier, Grégoire Massoullié, Pierre Ollitrault, Olivier Thomas, Raphaël Al Hamoud, Maria-Alexandra Nicola, Corentin Chaumont

**Affiliations:** Department of Cardiology, Rouen University Hospital, UNIROUEN, INSERM U1096, 1 rue de Germont, Rouen 76031, France; Department of Cardiology, Princess Grace Hospital, Monaco, Monaco; Department of Cardiology, Rouen University Hospital, Rouen, France; Department of Cardiology, Rouen University Hospital, Rouen, France; Department of Cardiology, Clinique Pasteur, Toulouse, France; Department of Cardiology, Grenoble University Hospital, Grenoble, France; Department of Cardiology, Grenoble University Hospital, Grenoble, France; Department of Cardiology, Clermont-Ferrand University Hospital, Clermont-Ferrand, France; Department of Cardiology, Caen University Hospital, Caen, France; Department of Cardiology, Clinique Ambroise Paré, Neuilly-Sur-Seine, France; Department of Cardiology, Rouen University Hospital, Rouen, France; Department of Cardiology, Princess Grace Hospital, Monaco, Monaco; Department of Cardiology, Rouen University Hospital, UNIROUEN, INSERM U1096, 1 rue de Germont, Rouen 76031, France

**Keywords:** Atrial fibrillation, Ablation, Personalized, Pulsed field ablation, AF mapping

## Introduction

In persistent atrial fibrillation (AF) recurrence rates remain high after pulmonary vein isolation (PVI) and additional strategies are debated^1,2,3^ and heterogeneously applied.^4^ Anatomical approaches such as posterior wall isolation (PWI) have not consistently improved outcomes,^5,6^ whereas personalized substrate-based strategies^7^ and the Marshall-PLAN^8^ have shown promise at the expense of extensive ablation and procedural complexity. A deterministic, physiology-driven algorithm (OCKHAM, Boston Scientific) integrated into the OPAL HDx mapping system identifies electrograms (EGMs) consistent with putative AF-sustaining regions, enabling a standardized personalized approach.^9,10^ We evaluated the feasibility, safety, and efficacy of an algorithm-guided pulsed field ablation (PFA) strategy in persistent AF (persAF) and compared its outcomes with a matched external cohort treated with PVI plus PWI using PFA.

## Methods

This prospective study included 50 consecutive patients undergoing first-time persAF ablation at two centres (March 2023–April 2025), all in AF at procedure onset. A detailed left atrial (LA) map was created during AF using a multipolar catheter (IntellaMap Orion), targeting recording of >30 000 points. EGMs consistent over at least three cycles (spatiotemporal and morphological similarity >60%) were analysed by the algorithm, which derived two parameters: local cycle length (L-CL), the local activation interval measured across the 64 electrodes of the mapping catheter, and duty cycle (DC), the percentage of the L-CL covered by electrogram activity across all catheter splines. Regions combining the shortest L-CL with DC >90% were highlighted as algorithm-identified, putative AF-sustaining regions. PVI and ablation of highlighted regions were performed with a pentaspline PFA catheter (Farawave 31 mm). Because the first non-navigated catheter version offered only rudimentary visualization on OPAL HDx, fluoroscopy was used to confirm catheter position over previously identified regions. PFA was delivered mainly in the flower configuration until complete regional coverage of the highlighted areas, avoiding gaps between targeted areas and/or anatomical boundaries. If AF persisted, remapping and further ablation of the new highlighted areas could be performed. AF termination was defined as direct restoration of sinus rhythm (SR) or organization into atrial tachycardia (AT) followed by AT ablation. The external comparator was drawn from the France PFA registry (NCT06497933) and restricted to patients with persAF who had undergone PVI plus PWI using the pentaspline PFA catheter alone. In all of them, PVI and PWI were confirmed at the end of the procedure using mapping and pacing from the pentaspline catheter. Propensity score matching (age, sex, AF type, LA dilation, rhythm at onset, CHA_2_DS_2_-VA) yielded a PVI + PWI reference group. Follow-up comprised 12-lead ECG and 24 h Holter at 4 months and 1 year, with additional testing if symptomatic; monitoring was identical in both groups. The primary endpoint was freedom from any atrial arrhythmia (>30 s) at 12 months after a single procedure, without a blanking period.

## Results

**Figure 1:**
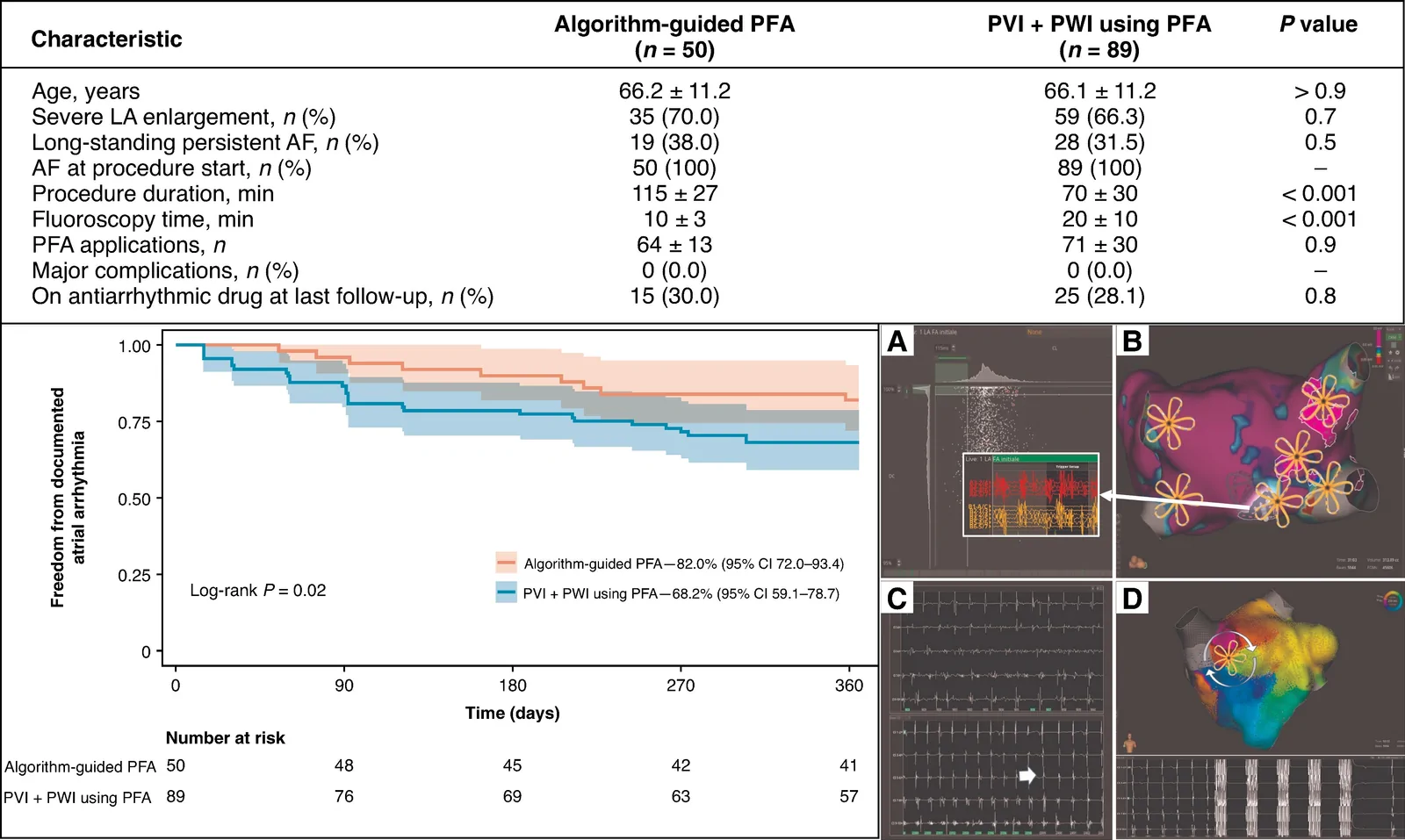
Baseline characteristics of the studied and matched population. Severe LA enlargement: diameter >50 mm and/or volume >40 mL/m^2^. Lower left panel. Kaplan–Meier estimates of 1-year freedom from any atrial arrhythmia according to ablation strategy (algorithm-guided PFA vs. PVI + PWI using PFA). Lower right panel. (*A*) Scatter plot of the consistent electrograms retained for OCKHAM analysis, with local cycle length on the *x*-axis and duty cycle on the *y*-axis. Electrograms with the shortest L-CL and highest DC were selected using green callipers (cut-offs: DC 95–100%, L-CL 115–141 ms); selected atrial electrograms at the targeted site are also shown. (*B*) LA 3D voltage map showing pulsed-field lesion sets (PVI + ablation of highlighted areas). (*C*) Coronary sinus recordings showing disorganized AF activity at the start of the procedure (upper) progressively organizing into an atrial tachycardia after ablation of all highlighted areas (arrow, lower). (*D*) Activation map of the atrial tachycardia (CL = 219 ms) showing rotational activity near the right superior pulmonary vein, where PFA (Farawave catheter, flower configuration) restored sinus rhythm, as confirmed on the coronary sinus recordings.

Patients were predominantly male (72%), aged 66 ± 11 years, with frequent LA dilation (92%); 38% had long-standing persAF. Mean procedure duration was 115 ± 27 min and fluoroscopy time 10 ± 3 min (*Figure 1*). The mean number of PFA applications was 64 ± 13 (50 ± 6 during PVI). A median of four regions was highlighted after the first map and two after the second; their distribution across nine LA regions was highly heterogeneous, the posterior wall being frequently but not systematically involved (30/50; 60%). A complete PWI was performed in 19 of these patients, whereas partial ablation of the posterior wall, specifically targeting the highlighted area, was performed in the remaining 11 patients. Peak L-CL increased progressively during ablation (171 ± 21 to 185 ± 25 ms; *P* < 0.001), reflecting slowing of the fibrillatory process. AF termination occurred in 17 patients (34%): direct SR restoration in 4 and organization into AT in 13. All patients with AT underwent ablation, resulting in restoration of SR. Among these, six patients required cavotricuspid isthmus ablation for typical atrial flutter that developed during the procedure. No major complications were observed in this small cohort.

During a median follow-up of 419 days, 46/50 patients (92%) were free of AF and 40/50 (80%) free of any atrial arrhythmia. Six patients presented with AT, including one with typical right atrial flutter. In the matched comparison, the algorithm-guided strategy was associated with higher 1-year freedom from atrial arrhythmia than PVI + PWI (log-rank *P* = 0.02; *Figure 1*). At last follow-up, 15/50 patients (30%) remained on antiarrhythmic drugs (amiodarone in 12), a proportion comparable to the matched cohort (25/89; 28.1%; *P* = 0.8).

## Discussion

In this first PFA-era evaluation of an algorithm-guided personalized strategy, targeting putative AF-sustaining regions was feasible and was associated with favourable 1-year rhythm control with a limited ablation burden and short procedure and fluoroscopy times. These outcomes were achieved in a challenging population entirely in AF at onset and with a high proportion of major LA enlargement. The heterogeneous distribution of highlighted regions supports an individualized approach and may help explain the inconsistent benefit of systematic PWI. The principal limitations are the modest sample size, the absence of right atrial mapping, and a rhythm-monitoring protocol relying on intermittent ECG and Holter that may underestimate asymptomatic recurrence despite being identical in both cohorts. Given the non-randomized design and likely residual confounding (operator experience, centre effect, procedural workflow), our findings warrant confirmation in randomized trials. Importantly, our study evaluates a combined procedural strategy (PVI + algorithm-guided regional ablation using PFA); outcomes cannot be attributed to the algorithm alone, which has not been independently validated as identifying causal AF drivers.

## Conclusion

In persAF, a personalized PFA strategy combining PVI with ablation of algorithm-identified regions was associated with higher 1-year arrhythmia-free survival compared with a systematic anatomical PFA approach in a matched external comparison.

## Data Availability

The data underlying this article will be shared on reasonable request to the corresponding author.
